# Does Dysphagia Predict Inpatient Morbidity and Mortality in Geriatric Patients Admitted for Aspiration Pneumonia?

**DOI:** 10.7759/cureus.39223

**Published:** 2023-05-19

**Authors:** Anmol Mittal, Mansi Patel, Daniel Wang, Ayham Khrais, Eric Tien Yen Chyn

**Affiliations:** 1 Department of Medicine, Rutgers University New Jersey Medical School, Newark, USA; 2 Department of Geriatrics, Mount Sinai Hospital, New York, USA; 3 Department of Geriatrics, Rutgers University New Jersey Medical School, Newark, USA

**Keywords:** microaspiration, presbyphagia, aspiration pneumonia, dysphagia in elderly, progressive dysphagia

## Abstract

Background

Aspiration pneumonia is common in older adults admitted for community-acquired pneumonia and is associated with significant morbidity and mortality. Factors that put this population at higher risk of aspiration include cognitive impairment, neuromuscular dysfunction, and dysphagia. This study aimed to determine whether a concurrent diagnosis of dysphagia conferred a higher risk of complications in the elderly admitted for aspiration pneumonia.

Methods

The National Inpatient Sample 2001-2013 database was queried for patients, aged 65 or older, with a diagnosis of aspiration pneumonia using International Classification of Diseases, Ninth Revision (ICD-9) codes. Sepsis, respiratory failure, and intubation were identified with their respective ICD-9 codes. A chi-square test and binary logistic regression analysis were used to examine socio-demographic and complication variables, with a significance level of α <0.001.

Results

A total of 1,097,325 patients were admitted for aspiration pneumonia, of which 349,861 (24.2%) had dysphagia. After incorporating socio-demographic variables, the dysphagia group had a significantly lower likelihood of having sepsis (OR=0.72), respiratory failure (OR=0.92), intubation (OR=0.52), and inpatient mortality (OR = 0.59). Patients with dysphagia had a significantly higher likelihood of increased length of stay (OR=1.24).

Conclusions

Elderly patients admitted with aspiration pneumonia with a co-diagnosis of dysphagia were less likely to have inpatient morbidity and mortality compared to their counterparts. This may be due to improved speech evaluation and treatment in patients with dysphagia allowing for better control of macro and micro aspiration. Future research is needed to examine if universal speech therapy can reduce hospitalization and long-term mortality for such patients.

## Introduction

Pneumonia is among the top 10 causes of death in the geriatric population and a frequent cause of exacerbation of chronic illnesses, and as such, imposes a significant economic burden [1]. The total annual excess cost of Medicare hospital-treated pneumonia in 2010 was over seven billion dollars [2]. Among cases of community-acquired pneumonia, 5% to 15% is accounted for by aspiration pneumonia (AP) [3]. Therefore, a comprehensive understanding of aspiration pneumonia is critical to improving healthcare well-being in older patients.

Aspiration pneumonia provides a unique challenge to the management of geriatric patients due to concerns for underlying dysphagia. Oropharyngeal dysphagia is a known risk factor for aspiration pneumonia. A study conducted in Spain detected oropharyngeal dysphagia via video fluoroscopy in 92% of patients admitted for pneumonia [4]. In addition, oropharyngeal dysphagia has been associated with an increased hospital stay as well as hospital readmission for pneumonia [5,6].

The pathophysiology behind oropharyngeal dysphagia leading to macro-aspiration and subsequent aspiration pneumonia involves a breakdown in protective mechanisms [7]. These mechanisms prevent the aspiration of ingested food, saliva, and foreign objects commonly introduced to the pharynx during feeding. Most notably, protective reflexes include the closure of the glottis and laryngeal anterior shift of the larynx [8]. Oropharyngeal dysphagia introduces a deficient response in these reflexes that can vary depending on the degree of dysphagia, which can be divided into seven degrees based on functionality [9]. Increasing degrees of oropharyngeal dysphagia lead to decreased pulmonary clearance mechanisms and dysfunctional response to foreign body aspiration [10]. This dysfunction includes decreased airway closure, lower expiratory peak flow, slower cough, and an extended swallow compression phase [7,11].

On the other end of the spectrum, oropharyngeal dysphagia and aspiration pneumonia have been linked with micro-aspiration as well. This frequently occurs during low levels of consciousness. A nuclear medicine study demonstrated aspiration during sleep in 71% of the geriatric patients with pneumonia versus 10% in control subjects [12,13]. The patients in this study had a high incidence of silent aspiration. The pathophysiology connecting oropharyngeal dysphagia, micro-aspiration, and aspiration pneumonia stems from a fundamental change in the lung microbiome. This altered microbiome combined with weakened pulmonary protective mechanisms, chronic use of proton pump inhibitors, and poor oral hygiene increase susceptibility to pneumonia in the elderly [12]. Of note, although dysphagia may be diagnosed and treated, micro-aspiration can still occur even when patients pass conventional screening tests such as the barium swallow and fiberoptic endoscopic evaluation of swallowing. This uncaught nightly aspiration then contributes to a breakdown of normal flora and immune response.

Presbyphagia is a possible mechanism for the increased prevalence of micro aspirations in elderly patients diagnosed with pneumonia. Presbyphagia involves a decrease in endocrine and exocrine function, loss of elasticity, changes in sphincter contractility, and sarcopenia as part of the aging process [14]. These decreases in function result in an increase in residual laryngeal bolus. Abrupt changes in hospital course including delirium and extended hospital stay may also precipitate episodes of dysphagia [14]. Through many of these age-related and iatrogenic changes involved in presbyphagia, elderly individuals experienced increased micro-aspiration.

Hence, all pneumonia in the elderly may have an aspiration component, whether it is on the macro versus micro aspiration spectrum. However, not all elderly pneumonia patients receive speech and swallow evaluation unless diagnosed with oropharyngeal dysphagia. Thus, screening for and the diagnosis of oropharyngeal dysphagia is especially important. Diagnosis tools include a barium swallow and transnasal video endoscopy, which provide an assessment of components of the swallow response and are able to see deficits in these reflexes [15]. Because oropharyngeal dysphagia commonly involves underlying issues, such as a malfunctioning neurological or musculoskeletal response, management entails addressing these issues following diagnosis. In those that are successfully found, interventions include swallowing exercises and dietary modifications specific to patients’ needs [16]. The use of appropriate feeding aid techniques, including neck extension and the Mendelsohn maneuver, helps prevent microaspiration and aspiration pneumonia [17].

This study aimed to determine whether a concurrent diagnosis of dysphagia conferred a higher risk of complications in the elderly admitted for aspiration pneumonia. We investigated how inpatient morbidity and mortality compare in patients with aspiration pneumonia with and without dysphagia. We hypothesized that patients with aspiration pneumonia with diagnosed dysphagia had fewer inpatient complications due to more robust evaluation and treatment for their dysphagia. Implications of correctly identifying this relationship could lead to more robust speech and swallow therapy for all elderly patients admitted for aspiration pneumonia. 

This article was previously presented as a meeting abstract at the 2021 American Geriatrics Society Annual Scientific Meeting on May 4, 2021.

## Materials and methods

Data source/study population

The National Inpatient Sample (NIS) database was utilized to perform a retrospective cohort analysis of inpatient admissions from 2001 to 2013. The NIS database is part of the Healthcare Cost and Utilization Project, which has been used in prior research as an effective tool to measure all-payor inpatient data. Data are collected from over seven million hospital stays each year that represent 20% of admissions from 47 states, including the District of Columbia, covering 97% of the U.S. population. Included in the data set are multiple elements such as admission diagnoses, procedures performed, length of stay, mortality events, and payor source. The database was queried for patients with a primary diagnosis of aspiration pneumonia (Online Resource 1). Age was stratified using 65 years of age as the minimum cut-off for the geriatric population. Institutional Review Board (IRB) approval was not obtained as the NIS database is de-identified.

Study variable/outcome

The study population was queried for the diagnosis of dysphagia. The primary study variables included common inpatient complications such as sepsis, urinary tract infections, myocardial infarction, respiratory failure, and intubation. Secondary analysis included the following variables: length of stay, insurance status (i.e. Medicaid vs Medicare vs Uninsured), race (Caucasian vs. African American vs. Hispanic vs. Asian/Pacific Islander/Native American), and genders assigned at birth (Male vs Female).

Statistical analysis

When analyzing patients admitted for aspiration pneumonia and the complications as they relate to a known diagnosis of dysphagia, a chi-square analysis was performed to determine variables to be included in a multivariable analysis with a 95% confidence interval. A binary logistic regression analysis was used to examine socio-demographic variables as well as complication and mortality variables with a significance level of p<0.001.

## Results

Patient characteristics and demographics

There were 1,785,941 patients admitted with a primary diagnosis of aspiration pneumonia, which equivalates to an estimate nationally of about 8,900,000 patients in a 13-year period or approximately 687,000 patients a year. Using the chi-squared analysis, there were significant differences between those with and without dysphagia between age, race, and median income groups (Table 1). There was no difference between the sex of patients. Of the patients in the sample with aspiration pneumonia, 411,503 (23.0%) were diagnosed with dysphagia. The incidence of the diagnosis of dysphagia in the patient population increased from 14% to 33%.

**Table 1:** Patient demographic data (N) *Significant level p<0.001

Variable	No dysphagia diagnosis	Dysphagia diagnosis	P-value
Race			< .001*
Caucasian	874,582	281,488	
African American	103,555	24,137	
Hispanic	63,578	21,995	
Asian, Pacific Islander, Native American	57,145	22,621	
Sex			< .001*
Males	715,689	230,528	
Females	658,659	180,952	
Insurance cohorts			< .001*
Private insurance	68,786	20,033	
Medicaid	20,640	5,767	
Medicare	1,263,916	380,205	
No insurance	4,784	1,266	
Respiratory failure			< .001*
No respiratory failure	113,0610	351,589	
Respiratory failure	243,829	59,914	
Intubation			< .001*
No intubation	128,4085	398,447	
Intubation	90,354	13,056	
Urinary tract infection			< .001*
No urinary tract infection	1,074,813	332,980	
Urinary tract infection	299,626	78,523	
Myocardial infarction			< .001*
No myocardial infarction	1,321,416	401,123	
Myocardial infarction	53,022	10,380	
Sepsis			< .001*
No sepsis	1,311,423	399,377	
Sepsis	63,016	12,126	
Mortality			< .001*
Alive	1,137,209	234,963	
Dead	370,193	40,798	
Length of stay			< .001*
Less than 5 days	627,756	172,629	
5 days or greater	746,598	238,838	

Prediction of complications in patients with dysphagia

**Figure 1:**
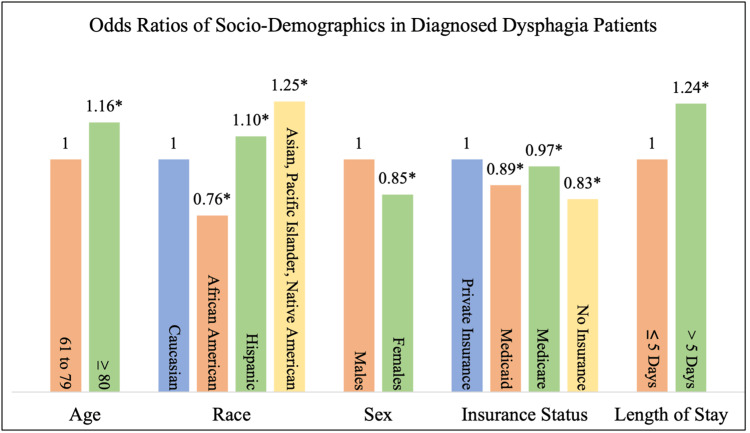
Odds ratios comparing socio-demographics in patients with and without diagnosed dysphagia *Significant at p<0.001

Using ages 65 to 79 as the reference, patients aged 80 and older were found to have a statistically significant difference in the occurrence of dysphagia (OR 1.16). When compared to Caucasian patients, every race except African Americans was more likely to be diagnosed with dysphagia. African Americans were 0.24 times less likely, Hispanic patients were 1.10 times more likely, and Asian, Pacific Islander, or Native American descents were 1.25 times more likely to have dysphagia. Females in the study were 0.15 times less likely to have dysphagia. Patients with all insurances other than private insurance were less likely to have dysphagia. Patients with Medicaid and Medicare were 0.11 and 0.03 times less likely to have dysphagia. Those that were uninsured were 0.17 times less likely. Patients with dysphagia had a significantly higher likelihood of a length of stay greater than five days (OR=1.24) (Table 2, Figure 1).

**Table 2:** Socio-demographic predictors of dysphagia patients *Significance level p<0.001

Variable	P-value	Odds ratio (95% CI)
Age		
65 to 79	Reference category	
≥ 80	.000^*^	1.16 (1.15-1.17)
Race		
Caucasian	Reference category	
African American	.000^*^	0.76 (0.74-0.77)
Hispanic	.000^*^	1.10 (1.09-1.12)
Asian, Pacific Islander, Native American	.000^*^	1.25 (1.23-1.27)
Gender		
Males	Reference category	
Females	.000^*^	0.85 (0.84-0.86)
Insurance status		
Private insurance	Reference category	
Medicaid	.000^*^	0.89 (0.85-0.92)
Medicare	.002	0.97 (0.96-0.99)
No insurance	.000^*^	0.83 (0.78-0.89)
Other insurance status	.000^*^	0.87 (0.83-0.91)

**Figure 2:**
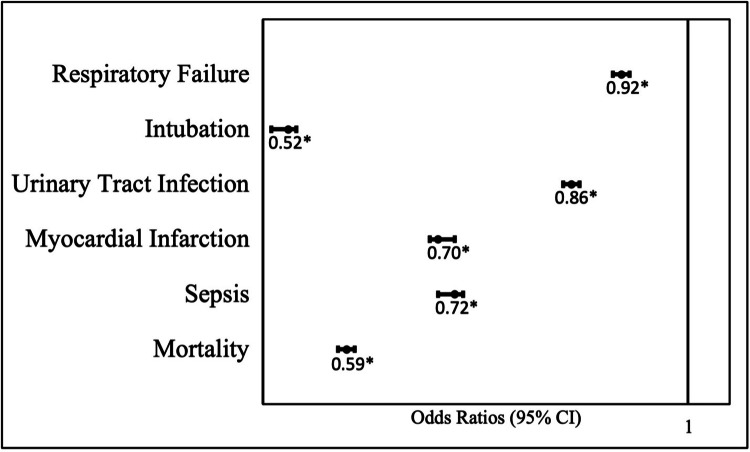
Odds ratios comparing complications in patients with and without diagnosed dysphagia *Significant at p<0.001

Patients with dysphagia were less likely to have sepsis (OR=0.72), urinary tract infection (OR=0.86), myocardial infarction (OR=0.70), respiratory failure (OR=0.92), and intubation (OR=0.52). Patients with dysphagia were also noted to have a significantly lower mortality rate compared to their counterparts (OR=0.59) (Table 3, Figure 2).

**Table 3:** Complication predictors in dysphagia patients *Significance level p<0.001

Variable	P-value	Odds ratio (95% CI)
Sepsis		
Not present	Reference category	
Present	.000^*^	0.72 (0.70-0.73)
Urinary tract infection		
Not present	Reference category	
Present	.000^*^	0.86 (0.85-0.87)
Myocardial infarction		
Not present	Reference category	
Present	.000^*^	0.70 (0.69-0.72)
Respiratory failure		
Not present	Reference category	
Present	.000^*^	0.92 (0.91-0.93)
Intubation		
Not performed	Reference category	
Performed	.000^*^	0.52 (0.50-0.53)
Mortality		
Alive	Reference category	
Dead	.000^*^	0.59 (0.56-0.60)
Length of stay >5 days		
Not present	Reference category	
Present	.000^*^	1.24 (1.23-1.235)

## Discussion

Dysphagia is an emerging geriatric syndrome that affects ~20% of independently living older individuals and ~30% of patients admitted inpatient [18]. It is associated with increased healthcare costs and a 40% increase in hospital length of stay [19]. Dysphagia is also associated with increased morbidity and mortality in hospitalized patients [20]. Given its significant healthcare impact, it is surprising to observe that the prevalence of a dysphagia diagnosis in our patient population admitted for aspiration pneumonia was merely 23% as compared to 92% reported by other studies [4]. Despite the increasing incidence rate of dysphagia diagnoses, we propose that dysphagia may be under-reported or underdiagnosed in most patients.

We found that patients admitted with aspiration pneumonia and a diagnosis of dysphagia had reduced inpatient morbidity and mortality. Patients diagnosed with dysphagia were seen to have a lower rate of sepsis, urinary tract infections, myocardial infarctions, respiratory failure, and intubation rates. This may be due to a combination of multiple factors. We theorize that a history of dysphagia suggests prior workup and evaluation by speech therapy, and therefore likely appropriate lifestyle and dietary modifications need to be made. Earlier and better chronic control of symptoms may contribute to less severe but increased length of stay of hospital admissions in these patients admitted for aspiration pneumonia [21]. Those with a pre-existing diagnosis of dysphagia may also present with symptoms of aspiration pneumonia earlier due to earlier caregiver awareness, allowing for better outcomes compared to those without a prior workup for dysphagia.

A reason why the diagnosis of dysphagia is commonly underreported is due to diagnosis coding variability [22]. However, it is important to elucidate other factors that may be contributing to the underdiagnosis of dysphagia. One such factor is clinician-provider awareness. For example, critical care providers are more likely to be sensitive toward oropharyngeal impairment in critically ill patients such as stroke patients or patients requiring intubation [23]. Providers who may not frequently care for patients more susceptible to dysphagia such as those patients with neurodegenerative conditions may be less likely to identify swallowing impairment and dysfunction. Dysphagia has also traditionally been seen as a black-and-white entity rather than as a continuum. Physicians do not evaluate for dysphagia as a daily routine examination nor is there a delineation between macro versus micro aspiration.

Provider variability in the awareness and identification of dysphagia is further exacerbated by the lack of a standardized and uniform screening protocol for dysphagia on both an inpatient and outpatient basis. A variety of tests are used to screen for dysphagia, including the three-ounce water swallow test (WST), bedside swallow assessment, Burke dysphagia screening test, and gugging swallow screen [24]. These modalities are commonly used to screen inpatients for dysphagia; however, this is not common practice in outpatient or residential care settings. A diagnosis of dysphagia may continue to remain unrecognized and thus underreported due to the absence of a well-established, gold-standard screening protocol for dysphagia. Given that stakes are high if dysphagia is underdiagnosed, pre-emptive speech therapy may be warranted for all elderly patients admitted for aspiration pneumonia.

Limitations

This study has several limitations. This was a retrospective study that relies on medical diagnoses and patient outcomes to be identified through medical billing codes. Coding is variable and physician dependent and thus the prevalence and incidence of dysphagia in patients admitted for aspiration pneumonia may be falsely low if coded under ICD codes different from those used to identify dysphagia in this study. Furthermore, we did not further delineate whether patients had oropharyngeal or esophageal dysphagia, both of which have different interventions and would likely carry different prognoses in terms of hospital course and adverse outcomes. Another factor to consider is how and when the diagnosis of dysphagia was made for those patients admitted for aspiration pneumonia, as having a long-standing history of dysphagia may also subject this study to lead time bias.

## Conclusions

Geriatric patients admitted for aspiration pneumonia with a co-diagnosis of dysphagia were associated with a prolonged, but less severe, hospital course and reduced mortality. It is difficult to assess from our database study whether patients were diagnosed with dysphagia prior to or during their admission or what the quality/standard of treatment they received was. What our study did show was a correlation that highlights the need for a future robust evaluation that can fully address whether hospital course morbidity/mortality is affected by a pre-admission dysphagia diagnosis.
